# Benznidazole affects expression of Th1, Th17 and Treg cytokines during acute experimental *Trypanosoma cruzi* infection

**DOI:** 10.1186/s40409-017-0137-4

**Published:** 2017-12-12

**Authors:** Mariana Gatto, Larissa Ragozo Cardoso Oliveira, Fernanda De Nuzzi Dias, João Pessoa Araújo Júnior, Carlos Roberto Gonçalves Lima, Eliana Peresi Lordelo, Rodrigo Mattos dos Santos, Cilmery Suemi Kurokawa

**Affiliations:** 1 Department of Tropical Diseases, Botucatu Medical School, São Paulo State University (UNESP - Univ Estadual Paulista), Av. Professor Mário Rubens Guimarães Montenegro, s/n, Distrito de Rubião Júnior, Botucatu, 18.6186-87 SP Brazil; 2 Department of Microbiology and Immunology, Botucatu Biosciences Institute, São Paulo State University (UNESP – Univ Estadual Paulista), Botucatu, SP Brazil; 3 Department of Biological Sciences, School of Pharmaceutical Sciences, São Paulo State University (UNESP – Univ Estadual Paulista), Araraquara, SP Brazil; 4 Department of Immunology, University of Western São Paulo (Unoeste), Presidente Prudente, SP Brazil

**Keywords:** Chagas disease, *Trypanosoma cruzi*, Immune response, Cytokines, *q*-PCR

## Abstract

**Background:**

The present study evaluated the effect of treatment with benznidazole on mRNA expression of IFN-γ, IL-17, IL-10, TGF-β and FoxP3 in spleen and heart tissue of BALB/c mice in the acute phase of an experimental infection with *Trypanosoma cruzi*, strains JLP or Y.

**Methods:**

The mRNA expression of cytokines and parasite load were assessed by *q*-PCR. Dependent groups were compared using Student’s paired t-test and independent groups were compared using Student’s unpaired t-test.

**Results:**

Infection with the JLP or Y strains increased expression of IFN-γ in the heart and of IL-10 and IL-17 in the spleen and heart compared to uninfected animals. Treatment increased the expression of IFN-γ and decreased the expression of IL-17, IL-10, TGF- β and Foxp3 in spleen and heart tissue compared to untreated infected animals.

**Conclusion:**

Benznidazole can induce Th1 profile in the initial of the acute phase. The treatment decreased the parasite load in both organs, although the number of parasites in Y-strain-infected mice remained high. The data suggest that benznidazole may modulate cytokine expression in infection and can be dependent of the strain. However, treatment was not fully effective in the infection provoked by Y strain, probably due to the characteristics of the strain itself.

## Background

Chagas disease (CD), an endemic infection caused by the protozoan hemoflagellate *Trypanosoma cruzi* (*T. cruzi*), is a major public health problem in Latin America with nearly 10 million infected individuals while a further 25 million are considered at risk [1–5].

The acute phase of infection is usually subclinical and with nonspecific symptoms [6]. In this phase, there is deep parasitemia and the host immune system works to isolate *T. cruzi* in an attempt to avoid dissemination [7, 8]. However, the inefficiency of the immune response in achieving complete elimination of the parasite ensures that *T. cruzi* persists in the host, which evolves to the chronic form of CD and may present different manifestations [9]. CD patients can remain years without developing clinical symptoms, thus characterizing the indeterminate phase of the disease or evolve to a symptomatic chronic phase with cardiac and/or digestive alterations [10–13]. Genetics, host immunity and parasite characteritics can result in different symptoms and clinical signs of CD [6, 14, 15].

Acute phase parasitemia control and development of the chronic phase are probably the result of parasite-host interaction that involves the cooperative action between drug effects and the host immunological response [16, 17]. Proinflammatory cytokine production, such as IL-12, IFN-γ and TNF-α, are required to activate T lymphocytes, macrophages and other cells, resulting in parasitemia control [7, 18–21]. Another cytokine that has been investigated in CD is IL-17. The IL-17 is related to protection, and high levels of this interleukin result in decreased parasitemia and increased production of inflammatory cytokines such as IFN-γ, IL-6 and TNF-α [22]. Furthermore, high IL-17 levels lead to less injury and lower mortality, a fact probably associated with its regulatory role in controlling the effects of other cytokines, such as IFN-γ and IL-12 [23]. Despite the publication of some studies that assessed IL-17, its role in CD remains unclear.

In addition to the pathogen elimination, the effector immune response uses different regulatory mechanisms to reduce tissue damage caused by excessive inflammation [24]. One of these mechanisms is performed by a subset of CD4^+^ T lymphocytes called regulatory T cells (Tregs), which produce TGF-β and IL-10, and also express the CD25 receptor and the transcription factor Forkhead BoxP3 (CD4^+^CD25^+^Foxp3^+^) [25]. Tregs are able to modulate the immune response to self-epitopes, tumoral cells and pathogens; however, it can excessively supress the immune response and impair the resolution of infection [26, 27]. The exact role of Tregs in the Chagas disease, mainly during treatment, is still unclear. A study showed that patients with the indeterminate form of CD have an increased frequency of CD4^+^CD25^high^, which produces high IL-10 and TGF-β levels, suggesting that Tregs contribute to the efficient control of parasite by effector cells without developing deleterious response and tissue lesions [28]. On the other hand, other studies have shown that Treg cells are not related to the immunopathogenesis of disease. Inactivation of Tregs cells resulted in low parasitemia and mortality of mice infected with *T. cruzi*, and did not affect the inflammatory response or frequency of TCD8^+^ cells in inflammatory foci [29, 30].

The treatment of CD is based on benznidazole (BZN), an effective drug during the acute and initial indeterminate chronic phases of infection and for congenital infection. However, its effectiveness in the phase of the disease is still unclear [31]. The drug can interfere directly in the synthesis of *T. cruzi* DNA, proteins and lipids, which facilitates the elimination of the parasite and affects iNOS gene expression, thus enhancing phagocytosis and modifying pro- and anti-inflammatory mediators to reduce the synthesis of IL-10, IL-1β, IL-6 and nitrite [32, 33]. The exact mechanism by which BZN acts remains unclear, but studies suggest that treatment in cooperation with the host immune system has a large impact on the clearance of parasites [17, 34].

Thus, we believe that the analysis of BZN treatment in relation to some aspects of the host immune response, such as pro- and anti-inflammatory cytokines, could better elucidate treatment impact on the acute phase of the infection by *T. cruzi* strains with different virulences. It would also provide a better understanding of the parasite-host interaction in CD. Therefore, this study aimed to evaluate the effect of BNZ treatment on mRNA expression of IFN-γ, IL-17, IL-10, TGF-β and Foxp3 in spleen and heart tissue of mice infected with either different strains of *T. cruzi* in the acute phase of CD infection.

## Methods

### Animals

Eight to ten-week-old female BALB/c mice were obtained from the breeding colony of the Department of Tropical Diseases (UNESP). All animals received sterile water and food ad libitum throughout the experiment. All the procedures involving animals and their care were conducted in conformity with the national and international guidelines and were approved by the Animal Ethics Committee of the Botucatu Medical School of UNESP (protocol number: FMB-PE-85/2010, CEUA-854/10).

### *T. cruzi* strains

Two different *T. cruzi* populations were used: the Y strain – considered highly virulent – and the JLP strain – isolated from a chronic chagasic patient treated at the Heart Institute of the School of Medicine of São Paulo University (USP). Both strains were kindly provided by Dr. Vicente Amato Neto, from the Tropical Medicine Institute of USP, and maintained at the Tropical Disease Research Laboratory of Botucatu Medical School (UNESP).

### Experimental groups

Mice were separated into eight groups (*n* = 5), namely: G1 – control JLP (uninfected, untreated); G2 – treated control JLP (uninfected, treated with BZN); G3 – infected with JLP strain, untreated; G4 – infected with JLP strain and treated with BNZ; G5 – Y control (uninfected, untreated); G6 – treated control Y (uninfected, treated with BNZ); G7 – mice infected with Y strain, untreated; G8 – mice infected with the Y strain and treated with BNZ.

### *T. cruzi* infection

The Y and JLP *T. cruzi* parasites were maintained in vivo through serially passages in BALB/c mice to ensure their virulence. Cardiac puncture was performed in previously infected animals and blood containing trypomastigotes was analized by optical microscopy. Parasite concentration was adjusted to 10^4^ parasites/mL in Neubauer chamber and the final volume was adjusted with sterile buffered saline. Thus, 100 μL of suspension containing metacyclic Y and JLP trypomastigotes forms from *T. cruzi* strains was inoculated intraperitoneally in experiment animals and distributed into groups G3, G4 (JLP) and G7, G8 (Y). Infection was using 5 μL of caudal blood samples and examined by optical microscopy at the beginning and end of the acute phase of infection [35]. To undergo the same stress as the infected animals, control groups G1, G2, G5 and G6 were inoculated with 100 μL of saline on day one of infection.

Parasitemia curves for Y and JLP *T. cruzi* strains used in the present study were previously defined by the research group. The Y strain is more virulent with an acute phase of 14 days and peak parasitemia at day 7 post-infection, whereas the JLP strain presents a 28-day acute phase and peak parasitemia at 14 days after infection [36].

### BNZ treatment

During the acute phase of infection for each *T. cruzi* strain, mice were treated daily (100 mg/kg) with BNZ (Rochagan®). BNZ pills were macerated and diluted in saline and each animal received the treatment by gavage. Animals infected with the JLP strain started treatment at day 7 post-infection (p.i.) and ended at day 28 p.i. (end of acute phase), thus lasting 22 days. Animals infected with the Y strain were treated from day 4 p.i. until day 14 p.i. (end of acute phase), totaling 11 days. Animals without infection and treated with BNZ (G2 JLP and G6 Y) were treated by the same procedure as their respective infected groups. To promote the same stress level as BNZ-treated animals, the groups G1, G3, G5 and G7 started and followed treatment with 100 μL of saline by gavage on the respective days of the BNZ treatment.

### Euthanasia of animals

Mice of all groups of the JLP strain (G1, G2, G3 and G4) were euthanized after the end of the 22 days of treatment (day 29 p.i.) and animals of all groups of the Y strain (G5, G6, G7 and G8) were euthanized after 11 days of treatment (day 15 p.i.). Euthanasia was performed using an excessive dose (0.2 mL) of anesthetic (Hypnol® 3%, Syntec, Brazil) intraperitoneally.

### Cardiac and splenic parasitism by qPCR

The spleen and heart were removed from mice 22 days after infection in groups G3 and G4, and 11 days after in G7 and G8. Total DNA was extracted from approximately 100 mg of each organ by the phenol-chloroform isoamyl alcohol method (Life Technologies, USA). Afer tissue samples were frozen in liquid nitrogen, they received 1 mL of lysis buffer (NaCl 4 M; Tris-HCl pH 7.5, 1 M; EDTA 0.5 M) and were crushed. Then, to the crushed mixture was added lysis buffer, proteinase K (InvitroGen, USA) and SDS 10% followed by overnight incubation in a water bath at 37 °C. Next, the mixture received more proteinase K and was incubated for two hours in a water bath at 37 °C. ANE buffer 5X (sodium acetate 1 M; NaCl 4 M; EDTA 0.5 M; SDS 10%) and phenol-chloroform isoamyl alcohol (25:24:1) (Life Technologies, USA) were added to the tubes and them centrifuged for 20 min at 5000 rpm at 4 °C. Supernatant was removed and transferred to another tube and the step described above was repeated. To the supernatant 10 mL of chloroform isoamyl alcohol at a 24:1 ratio (Life Technologies, USA) was added, which was centrifuged for 20 min at 5000 rpm at 4 °C. The supernatant was then transferred and supplemented with NaCl 4 M and 20 mL of cold absolute ethanol (Sigma, USA). The tubes were maintained at −20 °C overnight and then centrifuged for 45 min at 5000 rpm at 4 °C. The precipitate DNA was left at room temperature for two hours, dissolved in 250 μL of sterile milli-Q water, diluted to 100 ng/and stored at −20 °C. The DNA samples were read by spectrophotometer (NanoDrop, Thermo Scientific, USA) and only samples with 260/280 ratio higher than 1.8 were utilized. Relative quantification of *T. cruzi* DNA was performed using a standard curve based method for relative real-time PCR data processing [37] with a 7300 real-time PCR Systems (Applied Biosystems, USA) and Maxima SYBR Green qPCR Master Mix (Thermo Scientific, USA) containing 100 ng of DNA.

Each quantitative PCR (q-PCR) reaction was set in duplicate in a total of 20 μL each, which contained 0.2 mM of each forward and reverse primer, 1 μL of template gDNA, 10 μL of qPCR master mix and 8.2 μL nuclease-free water. In addition, a “no template” control in duplicate was included on each plate to prove that amplicon contamination was absent. PCR conditions were as follows: initial denaturation at 95 °C for 10 min and 40 cycles at 95 °C for 15 s and 60 °C for 60 s. Amplification of specific products was confirmed by a single melting curve profile generated at the end of each run. Standard curves were constructed by a serial ten-fold dilution with the DNA of a positive control tissue infected with *T. cruzi*. This positive sample received the relative value of 100 and the concentrations in all other tissues were normalized proportionately. Quantitative real-time PCR DNA analyses were performed using *T. cruzi* primers sequences available in the GenBank database (Table 1).

**Table 1:** Primer sequences

Gene	Primer sequences
IFN-γ	F 5’- AgA ggA Tgg TTT gCA TCT ggg TCA-3’ R 5’- ACA ACg CTA TgC AgC TTg TTC gTg-3’
IL-17	F 5’- ACC gCA ATg AAg ACC CTg AT-3’ R 5’- TCC CTC CgC ATT gAC ACA-3’
IL-10	F 5’- gCC AAg CCT TAT Cgg AAA Tg-3’ R 5’- CAC CCA ggg AAT TCA AAT gC-3´
TGF-β	F 5’ - AAC AAT TCC Tgg CgT TAC CTT – 3’ R 5’ - CTg CCg TAC AAC TCC AgT gA- 3’
Foxp3	F 5’- CCC Agg AAA GAC AGC AAC CTT −3’ R 5’- TTC TCA CCA CCA ggC CAC TTg −3’
GAPDH	F 5’ - CCT CgT CCC gTA gAC AAA ATg-3’ R 5’ - TgA Agg ggT CgT TgAT ggC-3’
*T. cruzi*	F- 5’-gCT CTT gCC CAC AMg ggT gC-3’ R- 5’-CCA AgC AgC ggA TAg TTC Agg-3’.

### Gene expression of cytokines by RT-qPCR

The spleen and heart were extracted from mice of groups G1, G2, G3 and G4 22 days after treatment (JLP strain) and from groups G5, G6, G7 and G8 11 days after treatment (Y strain). Approximately 100 mg of each organ was stored in an RNASafer (Applied Biosystems, USA) and total RNA was extracted using the TRIZOL® reagent (Invitrogen, Canada) according to the manufacturer’s instructions. The concentration of total RNA was determined by the absorbance values of samples at 260 nm and expressed as ng/μL. All samples showed absorbance of approximately 2.0. The cDNA was synthesized from 1 μg of total RNA using reverse transcriptase (ImProm-II™ Reverse Transcriptase System, Promega, USA). The reaction conditions were those described for DNA and primers used by Cezário et al. [38]. The relative concentration of IFN-γ, IL-17, IL-10, TGF-β and Foxp3 was obtained as described above after normalization with GAPDH. Quantitative real-time PCR mRNA analyses were performed using murine primer sequences available in the GenBank database (Table 1).

### Statistical analysis

Statistical analysis was accomplished using the Prism program v. 4.0. Dependent groups were compared using Student’s paired t-test and independent groups were compared using Student’s unpaired t-test. The significance level was set at 5% or the corresponding *p*-value.

## Results

### Treatment with BNZ reduces parasite load

**Fig. 1:**
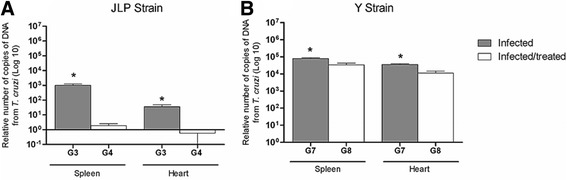
Parasite load of *T. cruzi*. Parasite load (DNA of *T. cruzi*) in the spleen and heart of BALB/c mice (*n* = 5) during acute infection with (**a**) JLP and (**b**) Y strains. G3; infected-JLP mice, G7; infected-Y mice, G4; infected-JLP mice and treated with BNZ, G8; infected-Y mice and treated with BNZ. Data are expressed as mean ± SD *p* < 0.05 G3 vs. G4 and G7 vs. G8

Our initial objective was to evaluate the treatment effects on parasite load in the spleen and heart of mice infected with either Y or JLP strain during acute infection. In relation to JLP strain (Fig. 1a), treatment significantly decreased the number of *T. cruzi* DNA in spleen and heart (G4) (mean ± SD: 1.893 ± 0.6916 and 0.59 ± 0.503 respectively) in comparison to spleen and heart of infected groups (G3) (1013 ± 230.4 and 35.85 ± 12.72 respectively). Smilar results were observed in Y strain (Fig. 1b). BNZ treatment significantly decreased number of *T. cruzi* DNA in spleen and heart (G8) (34,180 ± 0.8229 and 11,240 ± 3473 respectively) in comparison to spleen and heart of infected groups (G7) (79,520 ± 9363 and 35,360 ± 4282 respectively). However, it was observed that infection with Y resulted in more parasites in spleen and heart when compared with the JLP strain (Fig. 1a). Although treatment significantly decreased the number of *T. cruzi* DNA infecting both strains, this number remained high in Y strain infection, both in the spleen and heart (Fig. 1b).

### Treatment with BNZ increases gene expression of IFN-γ mRNA

**Fig. 2:**
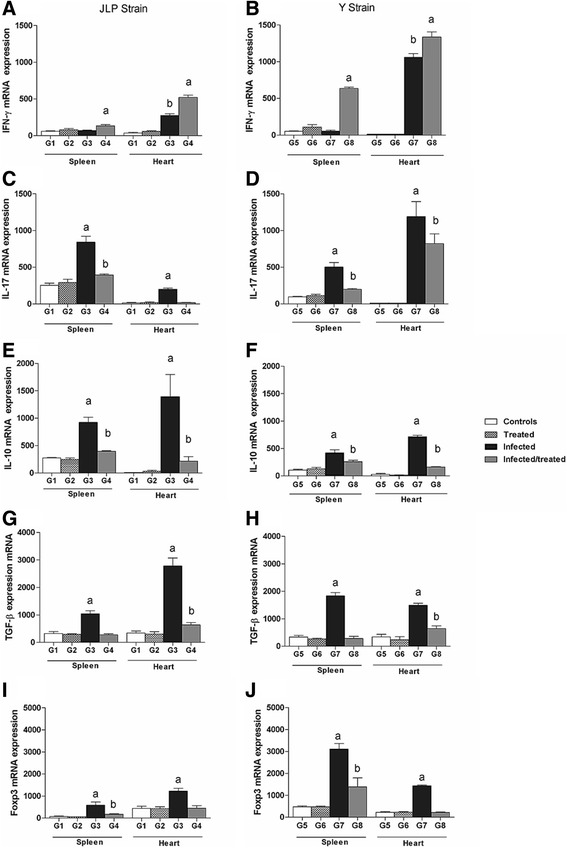
mRNA expression of IFN-γ, IL-17, IL-10, TGF-β and FoxP3. mRNA expression of IFN-γ, IL-17, IL-10, TGF-β and FoxP3 in the spleen and heart of BALB/c mice (n = 5) during acute infection with (**a**, **c**, **e**) JLP and (**b**, **d**, **f**) Y strains. G1, G5 – uninfected, untreated; G2, G6 – uninfected, BNZ-treated mice; G3, G7 – JLP-infected, untreated mice and Y-infected, untreated mice, respectively; G4, G8 – JLP-infected, BNZ-treated mice and Y-infected, BNZ-treated mice, respectively. Results are expressed as mean ± SD. *p* < 0.05. (**a**, **b**) **a –** G4 vs. G1, G2, G3 and G8 vs. G5, G6, G7; **b** G3 vs. G1, G2 and G7 vs. G5, G6. (**c-j**) **a** – G3 vs. G1, G2, G4 and G7 vs. G5, G6, G8; **b** G4 vs. G1, G2 and G8 vs. G5, G6

We assessed the effect of treatment on expression of this cytokine in the heart and spleen of animals infected with different strains of *T. cruzi* (Fig. 2). As to the spleen in JLP and Y strains, there was no significant difference among controls, treated and infected groups, but treatment significantly increased 1.9-fold IFN-γ mRNA in JLP infected/treated (G4) and 8.78-fold in Y infected/treated (G8) in comparison to other groups (G1, G2, G3 and G5, G6, G7, respectively) (Figs. 2a and b). As for the heart, the groups infected with JLP strain (G3) expressed 5.9-fold higher mRNA IFN-γ (*p* < 0.05) in relation to controls and treated groups (G1, G2). The BNZ treatmet (G4) increased 1.9-fold IFN-γ mRNA in comparasion to G3 and 11.3-fold IFN-γ mRNA in relation to G1 and G2 (p < 0.05) (Fig. 2a). The group infected with Y strain (G7) showed increasement of 197-fold IFN-γ mRNA (p < 0.05) when compared to G5 and G6. BNZ treatment (G8) increased significantly 1.26-fold mRNA in relation to G7 and 248-fold (p < 0.05) in comparasion to G5 and G6 groups (Fig. 2b).

### BNZ treatment decreases gene expression of IL-17, IL-10, TGF-β and Foxp3 mRNA

The respective treatment effects on IL-17, L-10, TGF-β and Foxp3 expression in the spleen and heart of animals infected with the Y or JLP strain are shown in Fig. 2. As to JLP and Y, the IL-17 expression did not differ between controls (G1, G5) and treated groups (G2, G6) in the spleen or heart. Infection with JLP (G3) strain significantly increased IL-17 mRNA expression in both spleen (2.7-fold) and heart (11.6-fold) in relation to other groups. Infection with Y strain (G7) significantly increased IL-17 expression in spleen and heart (3.6-fold and 4.33-fold, respectively) in comparasion to other groups (Fig. 2c and d). Infected/treated groups presented significantly decreased IL-17 mRNA in spleen (G4: 2.14-fold and G8: 2.52-fold) and heart (G4: 11.3-fold and G8: 1.45-fold) compared to infected groups (G3, G7). However, except for the heart in JLP strain, infected/treated groups showed higher IL-17 expression (*p* < 0.05) than control and treated groups (Fig. 2c and d).

We evaluated the effect of treatment with BNZ in IL-10, TGF-β and Foxp3 gene expression during the acute phase of infection. No significant differences were found in expression of IL-10, TGF-β and Foxp3 between control and treated groups (G1, G2 and G5, G6) in the studied organs. Mice infected with JLP strain (G3) showed significantly increased expression of IL-10, TGF-β and Foxp3 in spleen (3-fold, 3.5-fold and 5.8-fold, respectively) and heart (16-fold, 6.4-fold and 2.78-fold, respectively) compared to all other groups. Treatment in animals infected with JLP strain (G4) decreased significantly mRNA expression of IL-10, TGF-β and Foxp3 in spleen (2.33-fold, 3.76-fold and 3.5-fold, respectively) and heart tissues (6.43-fold, 4.38-fold and 2.73-fold, respectively) compared to infected groups (G3) (Fig. 2e–l).

Mice infected with Y strain (G7) showed significantly increased expression of IL-10, TGF-β and Foxp3 in spleen (2.55-fold, 6.2-fold and 4-fold, respectively) and heart (10.7-fold, 3.65-fold and 6.43-fold, respectively) compared to all other groups. Treatment with BNZ in mice infected with Y strain (G8) decreased significantly mRNA expression of IL-10, TGF-β and Foxp3 in spleen (1.62-fold, 6.51-fold and 2.24-fold respectively) and heart (4.39-fold, 2.31-fold and 6.73-fold) in relation to infected groups (G7) (Fig. 2f–j). However, IL-10, TGF-β and Foxp3 expression in infected/treated groups, with exception of TGF-β in the spleen (Fig. 2g, h) and Foxp3 in the heart (Fig. 2l, j), the mRNA expression remained higher (*p* < 0.05) in relation to control and treated groups.

## Discussion

BNZ has a cure rate of 86% in the acute phase of the disease, but only 8% in the chronic phase [39]. BNZ interferes in the synthesis of DNA, lipids and proteins of the parasites, facilitating their elimination [32]. Nevertheless, samples of *T. cruzi* naturally resistant to this drug could explain the low cure rate in some treated patients [40]. However, some studies report that activation of the immune system of the host seems to potentiate BNZ treatment efficacy in *T. cruzi* infection [16, 17, 34].

Our results show that treatment with BNZ in BALB/c mice with acute infection caused by the JLP or Y strain induced a significant reduction of parasites in the spleen and heart compared to infected animals that received no treatment. These results are in agreement with other experimental studies [41, 42]. It was observed that the number of parasites was considerably lower in JLP-infected than in Y-infected mice; and that the treatment led to the complete elimination of the parasite from the JLP strain. This difference may be because these strains of *T. cruzi* belong to different biodemes. The Y strain is characterized by a rapid multiplication and infection with high parasitemia, whereas the JLP strain seems to have slower multiplication and a later parasitemia peak [42, 43]. In addition, the different strains of *T. cruzi* presents variable behaviors, due to their distinct genetic and biological characteristics, which promote differences in virulence, tissue tropism, BNZ resistance and treatment efficacy [14, 44]. Moreover, distinct genetic characteristics of the host and parasite may be associated with different clinical forms of the disease [6, 14, 15].

In relation to IFN-γ, we observed that the heart of mice infected with Y and JLP strains presented greater IFN-γ expression in relation to uninfected groups. IFN-γ production in the early stages of CD is responsible for activating macrophages to produce reactive oxygen, thus inhibiting parasite replication [18, 20, 33, 45, 46]. *T. cruzi* in the myocardium triggers the immune response of type Th1. NK cells and *T. cruzi*-specific T lymphocytes migrate to heart and produce IFN-γ [47–49]. However, our results showed that in the spleen, IFN-γ expression did not differ between uninfected mice and those infected/untreated. Spleen cells produce low IFN-γ levels in early stages of infection, possibly due to low IL-12 production and low NK cells activity [50].

BZN alters immune response in acute phase and elicits the production of IFN-γ and possible differentiation of Th1 since our results demonstrated that treatment increased the mRNA expression of IFN-γ in infected groups, which is consistent with other studies that showed higher levels of IFN-γ after treatment [51]. On the other hand, a report showed that BNZ does not affect the production of IFN-γ in the serum of mice infected with the Y strain [41]. It is known that the most effective treatment and parasitological cure for the *T. cruzi* infection is associated with initial production of inflammatory cytokines in early stages of infection [19, 33, 51–54] The BNZ induces clearence of the parasite and triggers the release of antigens, which increases the production of IFN-γ and therefore enhances the action of the BNZ [55]. Production of IFN-γ probably has restored the immune response of type-Th1, leading to the decreas of parasites in tissue.

This study evaluated the effect of treatment on the IL-17 expression in experimental infection with either one of two *T. cruzi* strains of different virulences. IL-17 plays a crucial role in resistance to infection and absence of this cytokine in mice has increased *T. cruzi* in the liver, heart and kidney and decreased inflammatory cytokines such as IFN-γ, TNF-α and IL-6 [22]. Other studies have shown the importance of IL-17 in control of inflammation, in resolution of infection and parasite elimination [23, 56–58]. However, this citokine has been associated with inflammatory response and mortality in mice infected with *T. cruzi* [59, 60]. Our results indicate that in acute infection with the two strains, infected animals showed increased IL-17 expression, similarly to other studies [22, 23, 61, 62]. Further, higher levels of IL-17 were found in the heart in Y-strain infection compared to the JLP strain, probably due to the high parasite load and virulence of the Y strain, which may have been responsible for the stimulation and infiltration of a greater number of IL-17-producing cells.

On the other hand, treatment diminished the expression of this cytokine followed by a reduction in parasite load in both strains and organs studied. Similarly, Monteiro et al. [60] showed that survival of *T. cruzi-*infected mice was inversely proportional to the production of this cytokine. The IL-17 showed correlation with parasitemia load in spleen and heart tissue, the elevation of IL-17 in infected animals did not decrease the parasitemia load in tissue. However, the BNZ treatment restored the IL-17 produciton near to levels observed in control group, for both strains, suggesting non-involvement of IL-17 in protective mechanisms in this experimental model. The increase of IL-17 before treatment can balance the expression of IFN-γ, reforcing the decrease and favoring the parasite survival [54, 59].

Although several studies have shown the involvement of Treg cells in CD [28–30], the role of Tregs in CD treatment is poorly understood, mainly in the acute phase of the infection with distinct strains. In the acute phase, Tregs probably help to decrease parasitemia and balance inflammatory response [56, 63]. Understanding the role of Tregs could help to control the inflammatory response and tissue damage, to reduce the intense Th17 response and autoimune response observed in several infections [64, 65].

IL-10 e TGF-β are regulatory cytokines that possesses the ability to decrease inflammatory cytokines and appears to be deleterious in the early infection. In this way, these cytokines have been associated with susceptibility to *T. cruzi* infection [66, 67]. Our study showed that *T. cruzi* infection resulted in increased IL-10, TGF-beta and Foxp3 expression compared to controls. These results suggest that both strains of *T. cruzi* can induce Treg and cytokine production associated with this cells [63, 64].

The BNZ treatment decrease the expression of Foxp3, TGF-beta and IL-10. This decrease provokes IFN-γ production and Th1 profile. This findings suggest an efficient immune response of Th1 profile during the acute phase of infection a role in reducing parasitemia and reducing cardiac lesion. The bias of BNZ to reduce the lesion could be due IL-17 production; inflammatory cytokines associated with severity of DC and increased of neutrophils in cardiac tissue. The inflammation observed and described in DC increased the risk of morbidity in DC [64].

Newborns with CD produce higher amounts of IL-10 and lower amounts of IFN-γ than uninfected ones; and leukocytes of these patients fail to produce IFN-γ in vitro [68, 69]. According to our results, IL-10 significantly decreased after BNZ treatment, suggesting the involvement of this cytokine in mechanisms that suppress the immune response. Our results also suggest that IL-10 could be acting on the IFN-γ or other cytokines involved in protective mechanisms in infection with *T. cruzi* strains. The results also show that the two strains present the same response profile before and after treatment in the studied organs. Other studies showed that BNZ could modulate the synthesis of IL-10, altering the balance of cytokines and changing the course of infection [33, 70]. The treatment decreased the IL-10 expression, which may have contributed to the elevated expression of IFN-γ in infection with Y and JLP strains. However, another study showed that patients in the indeterminate phase, after undergoing the treatment with BNZ, presented a balanced immune response, with IFN-γ production by NK and T CD8^+^ cells, conferring effective treatment, and IL-10 production by CD4^+^ cells, responsible for clearance of parasites but without causing tissue damage or other deleterious effects during infection [16].

## Conclusions

Our results show that treatment with BNZ affects mRNA expression of IFN-γ, IL-17 and IL-10 in the acute phase of infection by different strains of *T. cruzi* in the spleen and heart. Although the strains respond similarly in relation to expression of these cytokines, the parasites of the Y strain were not eliminated. These results suggest that in addition to the beneficial effects of BZN in trypanocidal activity, which may vary according to the virulence and other strain characteristics, this treatment also displays immunomodulatory activity during infection. Additional studies are required to better understand the impact of BNZ treatment on the immune system and Treg modulation in infection with *Trypanosoma cruzi* strains with different virulence, especially in relation to the initial interaction of the parasites with cellular receptors and production of pro- and anti-inflammatory mediators and Tregs.

## Abbreviations

BZN: Benznidazole
CD: Chagas disease
P.i.: Post-infection
Treg: Regulatory T cell
